# The Hour of Confinement

**Published:** 1887-12

**Authors:** 


The Hour of Confinement.—A writer in *Le Journal de Medicine de Paris* has investigated this subject in *La Maternite Ste. Anne*, and finds that in each 1,000 cases, 450 occur between eight A. M. and eight P. M., and 550 between eight P. M. and eight A. M., the ratio being 100 to 122.

Dividing the day into quarters, cases occur as follows:

| From 6 A. M. to noon. | From noon to 6 P. M. | From 6 P. M. to midnight. | From midnight to 6 A. M. |
|---|---|---|---|
| 239 | 220 | 272 | 269 |
| 23.9 per cent. | 22. per cent. | 27.2 per cent. | 26.9 per cent. |

